# The first clinical case due to AP92 like strain of Crimean-Congo Hemorrhagic Fever virus and a field survey

**DOI:** 10.1186/1471-2334-9-90

**Published:** 2009-06-10

**Authors:** Kenan Midilli, Ayşen Gargılı, Onder Ergonul, Murat Elevli, Sevgi Ergin, Nesrin Turan, Gönül Şengöz, Recep Ozturk, Mehmet Bakar

**Affiliations:** 1 Microbiology and Clinical Microbiology Department, Istanbul University, Cerrahpaşa Medical Faculty, Istanbul, Turkey; 2 Parasitology Department, Istanbul University, Veterinary Faculty, Istanbul, Turkey; 3 Infectious Diseases and Clinical Microbiology Department, Marmara University, School of Medicine, Istanbul, Turkey; 4 Pediatrics Clinic, Haseki Education and Research Hospital, Istanbul, Turkey; 5 Virology Section, Pendik Veterinary Research Institute, Istanbul, Turkey; 6 Infectious Diseases and Clinical Microbiology Clinic, Haseki Education and Research Hospital, Istanbul, Turkey; 7 Infectious Diseases and Clinical Microbiology Department, Istanbul University, Cerrahpaşa Medical Faculty, Istanbul, Turkey; 8 Istanbul Branch of Ministry of Health of Turkey, Istanbul, Turkey

## Abstract

**Background:**

Crimean-Congo Hemorrhagic Fever (CCHF) is a fatal infection, but no clinical case due to AP92 strain was reported. We described the first clinical case due to AP92 like CCHFV.

**Methods:**

A case infected by a AP92 like CCHFV was detected in Balkanian part of Turkey. Diagnosis was confirmed by RT-PCR and sequencing. A human serologic and tick survey studies were performed in the region, where the case detected.

**Results:**

Thirty eight individuals out of 741 were found to be anti CCHFV IgM positive. The attack rate for overall CCHFV was calculated as 5.2%. In univariate analyses, CCHFV IgM positivity was found to be associated with the age (p < 0.001), male gender (p = 0.001), agricultural activity (p = 0.036), and history of tick bite (p = 0.014). In multivariate analysis, older age (OR: 1.03, CI:1.01–1.05, p < 0.001), male gender were found to be the risk factors (OR: 2.5, CI:1.15–5.63, p = 0.020) for CCHFV infection.

**Conclusion:**

This is the first human case with AP92 like CCHFV infection. Furthermore, this is the first report of AP92 like strain in Turkey. In the region, elderly males carry the highest risk for CCHFV infection.

## Background

The first Crimean-Congo hemorrhagic fever (CCHF) case in Turkey was reported five years ago [1]. The virus belongs to the genus *Nairovirus *in the *Bunyaviridae *family and causes severe diseases in humans, with a reported case fatality rate (CFR) of 3–30% [1]. By the year 2008, nearly 3000 CCHF confirmed patients with CFR of 5% were recorded at the Ministry of Health (MOH) of Turkey [2]. All of the confirmed cases, except one were detected in Anatolian region of Turkey.

The occurrence of CCHF closely approximates the known distribution of *Hyalomma *spp. ticks. Humans become infected through the bites of ticks, by contact with a patient with CCHF during the acute phase of infection, or by contact with blood or tissues from viremic livestock [3]. In a previous study, [4], all of the CCHF cases detected in Istanbul were imported cases, who had been infected out of Istanbul. Herein, we report a mild CCHF case from rural Balkanian part of Istanbul. Based upon this index case, we performed a serosurvey and field tick survey in the region, where the index case acquired the infection, and described the risk factors. In previous studies reported from Turkey, the CCHFV were belonged to the Europe-Turkey clade [4-6]. By this new report, we describe a newly emerged CCHFV strain in Turkey. Furthermore, this newly emerged strain that led to a mild case was not described as a human pathogen in the literature, previously [3].

## Methods

### Index case and the material

**Figure 1:**
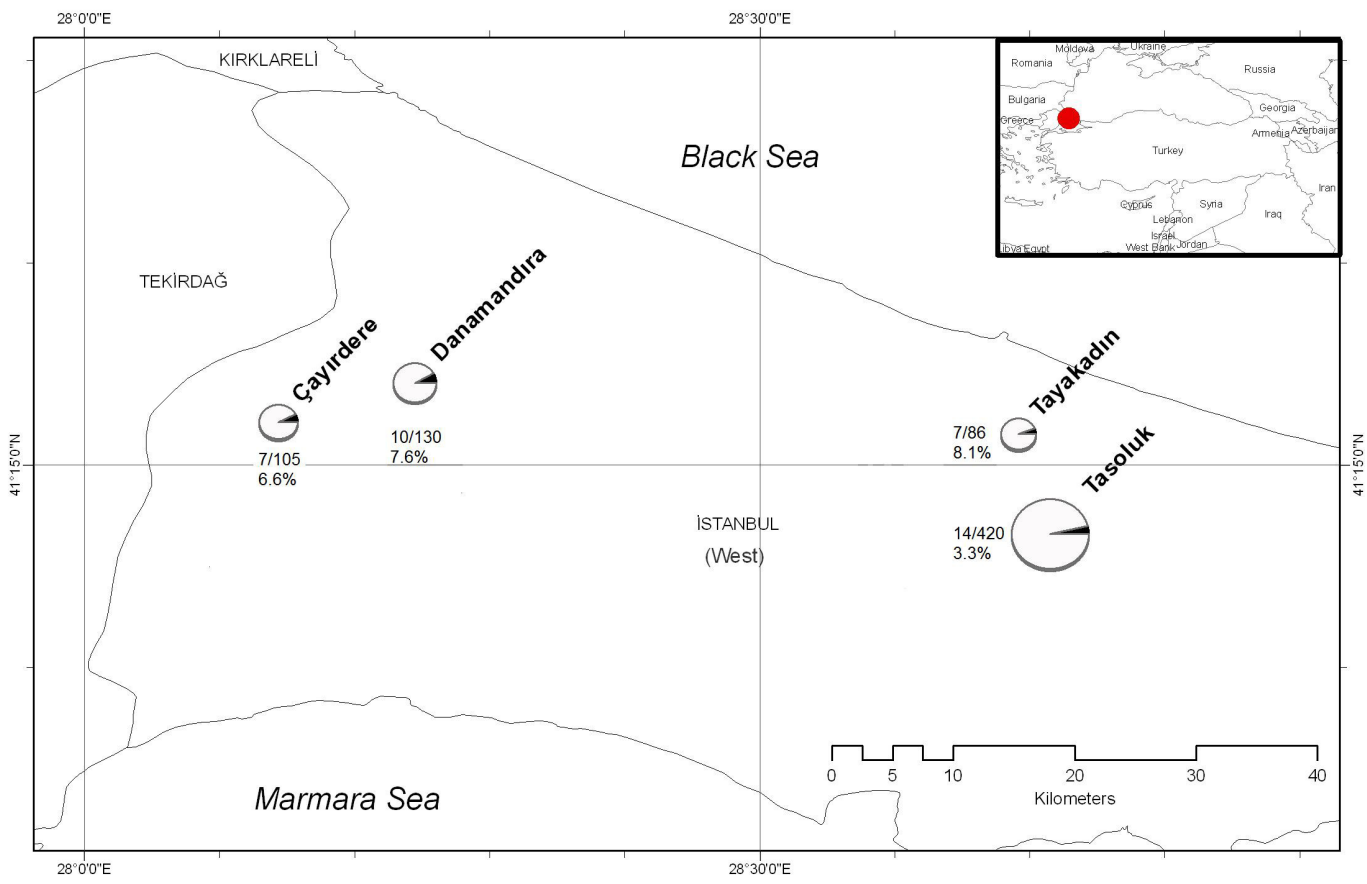
**Distribution of CCHF IgM positive individuals in the region**.

A mild CCHF case was diagnosed in a community hospital in Istanbul in June 2007. His disease started 3 days after the tick bite, and he was hospitalized 4 days after the tick bite. Serum sample was obtained 5 days after the tick bite was studied by RT-PCR for CCHFV RNA and by ELISA for IgG and IgM antibodies against CCHFV. After this case, a serosurvey and tick survey studies were performed in the region at the end of June 2007. The serosurveillance studies were performed in four major districts, which were depicted in figure 1. Seven hundred forty one subjects, who live in the region were surveyed. In total 56 ticks were collected from cattle from the same residential areas, and were screened for CCHFV RNA.

### Serologic studies

All the subjects were tested for IgM and IgG. A commercial variant of capture ELISA kit (vectorbest^®^, Russia) was used for detection of IgM antibodies against CCHFV. Immunoglobulin G antibodies were tested by ELISA kit of the same company (vectorbest^®^, Russia). The individuals, who were positive for IgM and/or IgG were prospectively surveyed, and four months later the second sera from these individuals were collected, and were studied for IgM and IgG. The initial sera were re-tested simultaneously with the second samples of sera. The informed consents from each individual were obtained.

### RNA extractions, PCR, and phylogenetic analysis

RNA was extracted from 200 μl whole blood using a commercial RNA extraction kit (High Pure Viral Nucleic Acid Kit, Roche Diagnostics^®^, Germany) and cDNA synthesized with Omniscript reverse transcription kit (Qiagen^®^, Germany) in accordance with the instructions of the manufacturer.

For designing of primers used during the diagnostic screening for CCHFV RNA, all S segment sequences from Turkey and some selected from eastern european countries were downloaded from the GeneBank and aligned using Clustal Wallis [7] software program. The sites of the possible primers were selected visually and evaluated using Primer test option of Primer Express software (Applied Biosystems^®^, USA). After initial diagnostic PCR, the amplicon was sequenced and the obtained sequence closely matched with the sequence of AP92 strain. For amplification of exclusively AP92 strain RNA we designed primers from S segment of this strain (Table 1).

**Table 1:** The primers, nucleotide numbers and the thercycling conditions

	Sequence	Position	Thermalcycling conditions		Amplicon lengths (bp)
Eecf-F1	ttg tgt tcc aga tgg cca gc	(49–68)*	95'C	2 min × 1	First round: 307
Eecf-R1	ctt aag gct gcc gtg ttt gc	(356–337)*	95'C	30 sec	
Eecf-F2	gaa gca acc aar ttc tgt gc	(115–134)*	60'C (57'C)#	1 min × 44	
Eecf-R2	aaa cct atg tcc ttc ctc c	(326–308)*	72'C	2 min	Second round: 211
			72'C	10 min × 1	

Gre-F1	aat gtg ccg aac ttg gac ag	(170–189)**	95'C	2 min × 1	First round: 593
Gre-R1	tgc gac aag tgc aat ccc g	(751–733)**	95'C	30 sec	
Gre-F2	atc aga tgg cca gtg caa cc	(198–217)**	57'C	1 min × 44	
Gre-R2	act ccc tgc acc act caa tg	(665–646)**	72'C	2 min	Second round: 469
			72'C	10 min × 1	

* According to the Hodhza strain (AY223475)

** According to the AP92 strain (U04958)

# In the second round

In both PCR assays 5.0 μl reverse transcription product was added to the 45 μl reaction mixture consisted of 1 μl forward primer (50 pmol/μl)) and 1 μl reverse primer (50 pmol/μl), 5 μl 10× reaction buffer, 3 μl 25 mM MgCl_2_, 1 μl dNTP stock (200 mM each dATP, dTTP, dCTP and dGTP) (Fermentas^®^, Lithuania), 1.25 U Taq DNA polymerase (Fermentas^®^, Lithuania) and 33.75 μl nuclease free water. For the second round amplification 2 μl first round products were added to the 48 μl reaction mixture with the same concentrations of the first round mixture. Thermal cycling conditions are given in Table 1. The amplification products were visualized following 1.5% agarose gel electrophoresis under UV-light.

The second round PCR products were cleaned up with a commercial PCR product purification kit (High Pure PCR Product Purification Kit, Roche Diagnostics^®^, Germany) and the purified products were subjected to the cycle-sequencing using big-dye terminator kit (ABI^® ^310, Foster City, Calif., USA). The excess primers and nucleotides were removed from cycle-sequencing products using sephadex-G50 fine columns, and the products were sequenced using *ABI*^®^*310 (Foster City, Calif.) USA *sequencer.

The obtained sequence was edited and aligned using Lasergene (DNA Star^®^) and Bioedit software packages [8]. For molecular analyses and comparisons, a 439 (between 218–657. nucleotides; numbering according to AP92 strain (U04958)) bp long portion of S segment was used. Phylogenetic analyses were carried out by distance method using neighbor joining algorithm with Treecon, version 1.3 b software [9]. Distances were calculated under Kimura 2 parameter model. Transition/transversion ratio was estimated from the data. Neither insertions nor deletions were taken into account. Topologic accuracy of the tree was evaluated by bootstrap method (1000 replicates) and only bootstrap values ≥ 70% were considered significant.

### Statistical analysis

Mean comparisons for continuous variables were done using independent groups *t *tests. Proportion comparisons for categorical variables were done using chi-square tests, although Fisher's exact test was used when data were sparse. A multivariate analysis was performed for the risk factors of IgM positivity. Age, gender, agricultural activity, dealing with husbandry, and history of tick bite were included to the model as independent variables. Significance was set at *p *< 0.05 using two-sided comparisons. STATA 10 (USA) software package was used in the analysis.

The study was approved by the Medical Ethics Committee of Istanbul Branch of Ministry of Health in Turkey.

## Results

### The index case

The index case was a 6 years old child. He applied to the emergency department of Haseki Education and Research Hospital in Istanbul with the complaints of fever, malaise, and the loss of appetite. He revealed the history of a tick bite 3 days ago, while he attended to a picnic in Tasoluk region of Istanbul. In his physical examination, the hyperemia in the site of tick bite was detected. In his laboratory investigation, alanine aspartate transaminase (AST) was found to be elevated (89 U/L, normal range: 5–42), activated partial thromboplastine time 43.7 second (normal range: 23–35 second), and prothrombin time was 20.1 seconds (normal range: 12–15 seconds). White blood cell count and platelet counts were found to be in normal limits. He was hospitalized for 10 days. The PCR for CCHFV RNA was found to be positive, and serologic tests for anti-CCHFV IgM and IgG by ELISA were found to be positive. The child was defined as a mild case, and discharged with total cure. His serum was obtained and retested by ELISA three months and one year later. His serum was found to be positive for CCHFV IgM and IgG antibodies three months later, and positive only for CCHFV IgG antibodies one year later.

The phylogenetic analysis of the obtained sequence of CCHFV revealed that, the strain was closely related to AP92 strain, which was reported from Greece. The sequence was deposited in gene bank under accession number of EU057975, and named as KMAG-Hu-07-01.

### Survey in the Region

In the first run, 39 (5.26%) subjects were found to be IgM positive, and 35 (4.72%) subjects were IgG positive. Thirty four out of 35 (97.4%) IgG positive patients were also IgM positive. Only one subject had IgG positivity, but IgM negativity (0.14%).

The second serologic analysis among IgM and/or IgG positives for CCHFV was performed 4 months later. Baseline sera were re-tested simultaneously for both IgG and IgM. All the IgM positivities except one, converted to IgG positivity. One IgG positive individual in the first run was found to be IgG negative in the second run. The first positive result of this individual was accepted as false, and this individual was considered as sero-negative. Accordingly, the analysis was performed among 38 IgM positive individuals versus 703 IgM negative individuals. These 38 IgM positive subjects were asked for the history of myalgia, fever, and flu like illness during summer months. Only one of the subjects had the complaints of myalgia and fever at the same time with the index case. This person attended to the same picnic with the index case.

The mean age was higher among IgM positive individuals (54 vs 37, p < 0.001). Among the IgM seropositive individuals, being older (54 vs 37, p < 0.001), male gender (76.3%, p = 0.001), performing agricultural activity (39.5%, p = 0.036), and having history of tick bite (15.8%, p = 0.014) were found to be significantly higher than IgM negative individuals (Table 2).

**Table 2:** Univariate risk factor analysis for IgM positivity

	IgM positives N = 38 (%)	IgM negatives N = 703	*P*
Mean age (sd)	54 (3.3)	37.2 (0.7)	< 0.001
Gender			
Male	29 (76.3)	341 (48.5)	0.001
Female	9 (23.7)	362 (51.5)	
Agricultural activity	15 (39.5)	171 (24.3)	0.036
Husbandry	13 (34)	163 (23.2)	0.120
History of tick bite	6 (15.8)	41 (5.8)	0.014

In multivariate analysis, IgM positivity was higher among older age (OR: 1.03, CI:1.01–1.05, p < 0.001) and males (OR: 2.5, CI:1.15–5.63, p = 0.020) (Table 3).

**Table 3:** Multivariate analysis for IgM positivity

	Odds ratio	Confidence interval	*P*
Age	1.03	1.01–1.05	< 0.001
Male gender	2.55	1.15–5.63	0.020
Agricultural activity	1.10	0.46–2.60	0.820
Husbandry	1.10	0.46–2.64	0.819
History of tick bite	2.34	0.88–6.22	0.086

Of the 56 ticks collected, 38 were identified as *Boophilus annulatus *and 18 were *Rhipicephalus bursa*. No CCHFV RNA was detected in ticks by RT-PCR.

## Discussion

**Figure 2:**
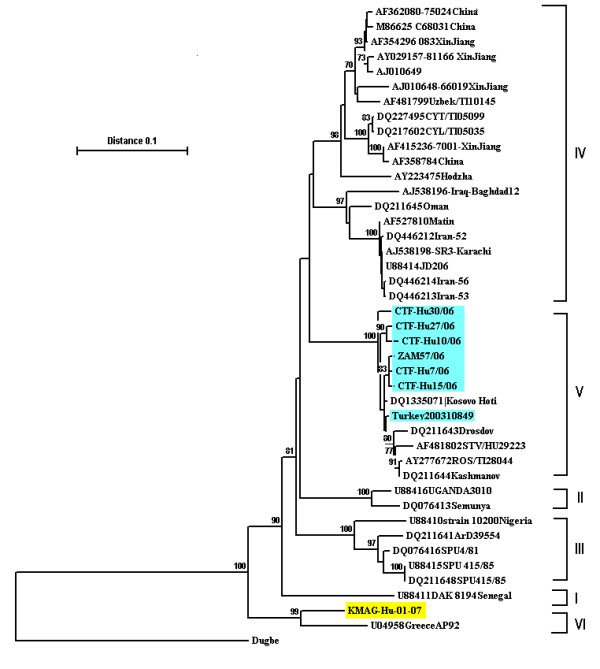
**Phylogenetic tree for the CCHF strains**. I: WestAfrica. II: Democratic. Republic of Congo. III: South/West Africa. IV: Asia/Middle. East. V: Europe/Turkey. VI: Greece (KMAG/hu/07/01 Tur: detected in this study).

This was the first CCHF case detected in rural Istanbul. This was a mild case according to the clinical and laboratory findings, which were described by Ergonul, et al [10]. The phylogenetic analysis of the partial S segment sequences of the strain, namely KMAG-Hu-07-01 revealed that it was closely related to AP92 strain reported from Greece [11,12] (Figure 2). AP92 strain differs from all known CCHFV strains (> 20% nucleotide difference), forming an independent phylogenetic clade [13]. The nucleotide sequence divergency between KMAG-Hu-07-01 strain and AP92 was 8.63% (38/440). However, at amino acid sequence level (146 amino acides) they differed only at two positions (1.36%). Previously reported strains either from Anatolian or European (Thrace) parts of Turkey were related to strains from Southern Russia and Balkan countries (Figure 2) [4].

The disease course of CCHFV infection among children was reported to be milder than adult patients [14]. The infection with AP92 strain was reported to be asymptomatic [15]. Accordingly, 4 individuals among 65 tested in the same region where AP92 strain was isolated in Greece, had antibodies against CCHFV without recalling any symptom resembling CCHF [15,16]. The results from a broad serosurvey study, which included 3,040 serum samples from apparently healthy residents from 26 of 54 counties in Greece, revealed an overall prevalence of 1.1% with a range from 0 to 6.3% [17].

The serosurvey studies for CCHFV infection in the region was very limited, but the transmission dynamics and the basic epidemiological measures such as the attack rate should be described [18]. By performing a serosurvey, we investigated the antibodies against overall CCHFV strains. In the region, 5.26% of the subjects had IgM positivity, and four months later all these IgM positive subjects, except one, developed IgG positivity. The IgM positivity implies the new infection. Except the presented case, the rest did not seek medical advice, and these patients were considered as asymptomatic cases. The attack rate for overall CCHFV infection, that was defined as the proportion of diseased subjects among the infected ones was two out of 38 (5.2%). The attack rate was very low compared to the CCHFV strains reported from Russia, where the attack rate was described as 20% previously [14]. One of the possible explanations for this difference could be the different virulent strains of CCHFV, but further studies are needed.

The region, where the survey was performed was the closest counties to the place, where the index case was attacked by the tick (Figure 1). In univariate analysis, age (p < 0.001), male gender (p = 0.001), agricultural activity (p = 0.036), and history of tick bite (p = 0.014) were found to be associated with IgM positivity. In multivariate analyes, IgM positivity was higher among older age group (OR: 1.03, CI:1.01–1.05, p < 0.001) and males (OR: 2.5, CI:1.15–5.63, p = 0.020) (Table 3). Being older and being male could be the predisposing factors for the exposure to the ticks, because of the increased working time in the field.

In previous studies, *Hyalomma marginatum*, which is the main vector of CCHFV was detected as the most frequent tick in Anatolia. However, the limited number of ticks collected in the region were typed as *Boophilus annulatus *and *Rhipicephalus bursa*. Further studies with larger numbers of ticks are necessary to describe the associaton between the tick species and the CCHV infection.

We couldn't isolate this strain, because of the lack of laboratory facilities with appropriate biosafety level. However, since this strain could be the agent for asymptomatic infection, its isolation and further characterization could be useful for undestanding the pathogenetic mechanisms and for vaccine development against CCHFV.

## Conclusion

A mild CCHF case from rural Balkanian part of Istanbul, who was infected by AP92 like CCHFV strain, which is new in Turkey was described. The strain was detected only from Greece previously, and was reported as the cause of asymptomatic infection. The attack rate for overall CCHF infection was very low (5.2%) in the region. The infection was more common among the males and older people, who work in the field.

## Competing interests

The authors declare that they have no competing interests.

## Authors' contributions

KM: Study design, laboratory work. AG: Study design, laboratory work, data collection. OE: Interpretation of data, data analysis, manuscript preparation. ME: Clinical diagnosis and follow up of the case. SE: Laboratory work. GS: Data collection. RO: Interpretation of data. MB: Data collection, interpretation of data.

## Pre-publication history

The pre-publication history for this paper can be accessed here:

http://www.biomedcentral.com/1471-2334/9/90/prepub
